# Follicle‐stimulating hormone impairs dental pulp stem cells odontogenic differentiation

**DOI:** 10.1111/jcmm.15681

**Published:** 2020-07-28

**Authors:** Hua Qian, Xiaoyue Guan

**Affiliations:** ^1^ Department of Tissue Engineering and Regeneration, School and Hospital of Stomatology, Cheeloo College of Medicine Shandong University & Shandong Key Laboratory of Oral Tissue Regeneration & Shandong Engineering Laboratory for Dental Materials and Oral Tissue Regeneration Jinan Shandong China; ^2^ Department of Endodontics College of Stomatology Xi'an Jiaotong University Xi'an China

**Keywords:** dental pulp stem cells, follicle‐stimulating hormone, menopause, odontogenic differentiation, reparative dentin

## Abstract

In addition to bone, the dentin‐pulp complex is also influenced by menopause, showing a decreased regenerative capacity. High levels of follicle‐stimulating hormone (FSH) during menopause could directly regulate bone metabolism. Here, the role of FSH in the odontogenic differentiation of the dentin‐pulp complex was investigated. Dental pulp stem cells (DPSCs) were isolated. CCK‐8 assays, cell apoptosis assays, Western blotting (WB), real‐time RT‐PCR, alkaline phosphatase activity assays, and Alizarin Red S staining were used to clarify the effects of FSH on the proliferation, apoptosis and odontogenic differentiation of the DPSCs. MAPK pathway‐related factors were explored by WB assays. FSH and its inhibitor were used in OVX rats combined with a direct pulp‐capping model. HE and immunohistochemistry were used to detect reparative dentin formation and related features. The results indicated that FSH significantly decreased the odontogenic differentiation of the DPSCs without affecting cell proliferation and apoptosis. Moreover, FSH significantly activated the JNK signalling pathway, and JNK inhibitor partly rescued the inhibitory effect of FSH on DPSC differentiation. In vivo, FSH treatment attenuated the dentin bridge formation and mineralization‐related protein expression in the OVX rats. Our findings indicated that FSH reduced the odontogenic capacity of the DPSCs and was involved in reparative dentinogenesis during menopause.

## 1 INTRODUCTION

Menopause is associated with many complications in females due to changes in hormones.^1^ In the past few decades, the cause of menopause has been attributed only to the decline in oestrogen levels.^2^ Oestrogen deficiency can not only contribute to osteoporosis but also lead to mineralization damage and reduced regeneration capacity of the dentin–pulp complex. These pathological changes are closely related to a series of changes in hormone levels caused by ovarian decline. One of the ways to achieve this effect is to regulate related tissue‐specific stem cells, including stem cell formation, stem maintenance, proliferation rate and differentiation.^3^, ^4^

Dental pulp stem cells (DPSCs) were first isolated and characterized by Gronthos et al from third molars and have become a great potential tool for tissue engineering.^5^ As a kind of somatic stem cells in dental pulp tissue, DPSCs can be efficiently obtained from medical waste (such as orthodontic teeth to be removed and impacted wisdom teeth) and have high proliferative, self‐renewal and multi‐directional differentiation ability. It is well known that DPSCs can differentiate into odontoblast‐like cells after mineralization induction in vitro.^6^, ^7^ The characteristics of DPSCs that can form dentin‐pulp complexes provide theoretical basis for the physiology, pathology and mechanism of dentin formation.^8^ It also reveals potential and advantages of DPSCs in the clinical application prospects of preservation and regeneration of pulp. Researchers found that oestrogen deficiency can reduce the odonto/osteogenic differentiation of DPSCs and impair the odontogenic capacity of the dentin‐pulp complex.^9^, ^10^, ^11^

The oestrogen level is controlled by the hypothalamus‐pituitary‐ovary axis and is mainly regulated by follicle‐stimulating hormone (FSH). FSH belongs to the hypothalamic hormone family and is a glycoprotein hormone secreted by the anterior pituitary. Recently, the traditional view that FSH works solely as a gonadal hormone has changed.^12^ More and more evidences showed that ovarian failure in late menopause is related to a sharp increase of serum FSH, which is consistent with the most rapid bone loss and the occurrence of visceral adiposity.^13^, ^14^, ^15^ A series of studies have shown that FSH directly regulates bone mass in mice via a non‐classical pathway by regulating osteoclast activity.^16^ FSHR, a specific receptor of FSH, was expressed in mesenchymal stem cells but not in osteoblasts and their precursors. A previous study showed that blocking FSH β not only inhibited osteoclast activity and reduced the inflammatory response but also increased bone formation and osteoblast number.^17^ This study suggested that FSH may play a role in inhibiting bone formation by inhibiting the differentiation of mesenchymal stem cells into osteoblasts. DPSCs/odontoblasts are similar to BMMSCs/osteoblasts in many aspects. Therefore, FSH may negatively regulate the odontogenic differentiation of DPSCs, impairing the dentinogenic capacity of the dentin‐pulp complex. The purpose of our research is to examine the effects of FSH and uncover the possible mechanism of the mineralization ability of DPSCs.

## 2 MATERIALS AND METHODS

### 2.1 DPSC isolation, culture and passaging

With the informed consent, healthy human third molars were collected from patients (18‐25 years old) at the Oral and Maxillofacial Surgery Clinics of Shandong Provincial Stomatological Hospital for orthodontic needs. Ethical approval was obtained from the Ethics Committee of the School of Stomatology, Shandong University, China. As previously described, the teeth were rinsed by phosphate buffered saline (PBS) with 2% penicillin and streptomycin, and the pulp was completely removed in a sterile clean biological safety cabin. The pulp was then cut into small pieces and digested in enzyme mixture (4 mg/mL trypsin (Gibco, Life Technologies) +3 mg/mL collagenase type I (Gibco, Life Technologies, Grand Island, NY, USA) for 50 minutes at 37°C. Equal volumes of neutralization medium (10% FBS in α‐MEM with 1% penicillin/streptomycin) were added to the digestive solution and mixed well to terminate the digestion. The dissociated‐cell solution was then centrifuged at 1000 rpm for 5 minutes to collect the cell precipitates. Subsequently, the cell precipitates were cultured in α‐MEM (Hyclone) containing 20% foetal bovine serum (FBS; BI technology), 100 U/mL penicillin and 100 μg/mL streptomycin in a 37°C incubator with 5% CO_2_. After reaching 80%‐85% confluence, The DPSCs were subcultured. The cells at 3rd–5th passages were used in next studies.

### 2.2 Flow cytometry (FCM)

For DPSC characterization, cells in log phase were digested, resuspended, counted and incubated with each surface molecular markers antibody according to the antibody instructions at 37°C in dark for 1 hour. After washing three times with 0.01 mol/L PBS, the expression of each surface molecule was examined by a BD FACSCalibur system (BD Biosciences, San Jose, CA, USA). The following antibodies were used: CD34‐APC, CD45‐APC, CD44‐PE, CD90‐FITC, CD105‐APC, CD29‐PE, CD146‐PE (BD Biosciences, San Jose, CA, USA) and Stro‐1‐FITC (R&D Systems, Minneapolis, MN, USA).

Apoptosis characteristics of cells were also detected by flow cytometry. After 24 hours treatment with FSH at concentrations of 0, 3, 10, 30 and 100 ng/mL, DPSCs were trypsinized, collected, washed twice with PBS and detected by Annexin V‐FITC‐PI Apoptosis Detection Kit (BD Biosciences, San Jose, CA, USA) following the manufacturer's instructions. All experiments were repeated three times.

### 2.3 Cell counting kit‐8 assay (CCK‐8)

DPSCs (2 × 10^3^ cells/well) were plated on 96‐well plate. To detect the effect of FSH on the proliferation of DPSCs, cells were incubated with CCK‐8 reagents (Dojindo, Kyushu Island, Japan) at 37°C for 2 hours at the indicated time points for CCK‐8 analysis. Optical density (OD) at 450 nm was detected by a microplate reader (Bio‐Tek, Winooski, VT).

### 2.4 Odontogenic differentiation

DPSCs (1 × 10^4^ cells/well) were seeded on six‐well plates (Eppendorf) and cultured until the cells reached 80% fusion. 2 mL of odonto‐osteogenic medium (α‐MEM containing 10% FBS, 100 μmol/L L‐ascorbic acid, 10 mmol/L β‐glycerophosphate and 10 nmol/L dexamethasone) with FSH at concentrations of 0, 3, 10, 30 and 100 ng/mL was added to the cultured cells for odontogenic induction.

### 2.5 Alkaline phosphatase (ALP) activity

DPSCs were cultured in odontogenic medium with FSH and SP600125 for 7 days, and the medium was changed every other day. Then, they were washed 3 times with PBS and incubated with 50 μL of 0.2% Triton X‐100 at 4°C overnight. After confirming that the cells were completely lysed under microscope, ALP activity were measured by corresponding kit (Jiancheng, Nanjing, China) referred to the manufacturer's instructions. Optical density (OD) was determined at 450 nm and normalized to total protein by a BCA kit (Beyotime, Shanghai, China).

### 2.6 Mineralized nodule formation and detection of calcium content

The mineralization ability of the DPSCs treated with FSH at concentrations of 0, 3, 10, 30 and 100 ng/mL in the odontogenic induction group and non‐induced group was investigated by Alizarin Red S staining. DPSCs in 6‐well plates (Eppendorf) were cultured in odontogenic medium and FSH at each concentration. After two weeks, the cells were washed twice with PBS and fixed in 4% paraformaldehyde for 15 minutes at room temperature. Then, the cells were washed twice with deionized water and stained with 100 μmol/L Alizarin Red (pH = 4.2, Sigma‐Aldrich) for 2 minutes. Staining of the mineralized nodules was recorded under light microscope. Afterwards, 10% cetylpyridinium chloride (CPC, Sigma‐Aldrich) in 10 mmol/L sodium phosphate was applied to dissolve Alizarin Red S at 25°C for 30 minutes. The final calcium concentrations were obtained by detecting absorbances at 560 nm and were normalized to the total protein concentrations.

### 2.7 Real‐time transcription polymerase chain reaction (PCR)

Total RNA was extracted from DPSCs treated with FSH and odontogenic medium for 3 days performed with the MiniBEST Universal RNA Extraction Kit (TaKaRa, Dalian, China) and quantified with Nanodrop 2000 (Thermo Fisher Scientific, Waltham, MA, USA). cDNA was synthesized using Primescript RT Master Mix kit (TaKaRa). Real‐time RT‐PCR was performed with the SYBR Premix Ex Taq kit (TaKaRa) in Roche real‐time PCR system. Primers sequences for amplifying FSHR, RUNX2, ALP, DSPP and GPADH mRNA expression levels were presented as supplementary data (Table S1). The quantitative PCR conditions were set as the manufacturer's instructions. The mRNA expression levels were calculated by the 2^−ΔΔCt^ method. All PCRs were carried out in triplicate.

### 2.8 Western blot

For analysis of the expression of odontogenic‐related proteins, total DPSCs induced after 7 days were extracted. For MAPK pathway‐related proteins, DPSCs were cultured in serum‐free medium for 24 hours and then treated by FSH (30 ng/mL) for 0, 30, 60, 90 and 120 minutes, respectively. Total protein of DPSCs at 0, 30, 60, 90 and 120 minutes of treatment with FSH (30 ng/mL) were harvested. For analysis of the effects of different concentrations of FSH on the odontogenic differentiation of DPSCs and FSHR, the DPSCs were collected at day 7. After washing three times with PBS, the DPSCs were lysed in RIPA buffer (Beyotime, Shanghai, China). Equal amount of protein was loaded to 10% sodium dodecyl sulphate‐polyacrylamide gel electrophoresis and transferred to polyvinylidene fluoride (PVDF, Millipore, Darmstadt, Germany) membranes. Then, the membranes were blocked in 5% BSA at room temperature for 1 hour and incubated with primary antibodies overnight at 4°C. After incubation with relative secondary antibodies at 4°C for 1 hour, and the antibody‐antigen reaction was detected using a Western Blotting Imaging System. The primary antibodies used in this paper included DSPP (Solarbio, Littleton, USA), ALP, RUNX2, OSX (Abcam, Cambridge, UK), FSHR (Sigma‐Aldrich), p‐ERK, ERK, p‐P38, P38, p‐JNK and JNK (Cell Signaling Technology), and GAPDH (ProteinTech, Wuhan, China).

### 2.9 Animal experimental groups

Forty female Sprague Dawley rats of 12‐week‐old weighing 180‐200 g were purchased from the Laboratory Animal Center of Shandong University. The experimental protocol was approved by the Institutional Animal Care and Use Committee of Shandong University. Experimental rats were randomly assigned to 4 groups based on our previous study: group 1, sham surgery + 0.1 PBS(pH 7.4) as a vehicle; group 2, bilateral ovariectomy (OVX) + vehicle; group 3, bilateral OVX + 1.6 mg/kg Leuprorelin (LE, Takeda Pharmaceutical Company, Ltd., Osaka, Japan); and group 4, bilateral OVX + 3 µg/kg FSH (Merck).^18^

### 2.10 Ovariectomy for the rats and drug application

The rats were anesthetized via the intraperitoneal injection performed with 10% chloral hydrate (Qilu hospital, Jinan, China). Bilateral ovariectomies were performed in the rats of the OVX group, while rats in the sham group were subjected to sham surgeries. After surgery, the rats of the corresponding groups were injected subcutaneously with LE, FSH or vehicle at relevant concentrations. LE is a sustained‐release dosage form that maintains effective blood concentration and prevents circulating FSH levels increase for at least 4 weeks after injection. (Filicori et al, 1996).

### 2.11 Direct pulp capping

One week after the ovariectomies and sham surgeries, all rats were anaesthetized as described earlier. The direct pulp capping of rats were performed as Tran et al reported.^19^ Briefly, after chemically cleaned and disinfected by NaOCl (5%) and 0.1%, Chlorhexamed Fluid (Evonik‐Degussa, Germany), the cavities were prepared on the mesial surface of maxillary first molars under sterile water cooling. When it is close to the pulp, 10# K file was used to induce perforation of pulp chamber. The perforations were directly capped with white ProRoot MTA cement (Dentsply Tulsa Dental, Tulsa, OK, USA) and filled with GC Fuji IX glass ionomer cement (GC Corporation, Tokyo, Japan) following cleaning with sterile saline and paper points.

### 2.12 Detection of serum hormone levels

Blood samples were collected from all rats on the 7, 14 and 21 days after pulp capping through the groin vein. The serum was separated from blood samples by centrifuging at 1000 g for 10 minutes under 4°C and measured within 48 hour to detect the levels of E2 and FSH according to previous reports.^20^

### 2.13 Tissue preparation

On day 21 after direct pulp capping, the rats were killed. The samples were dissected and fixed with 4% paraformaldehyde for 2 days at 4°C and demineralized in 10% EDTA for at 4°C for approximately 4 weeks. The specimens were dehydrated, clarified, embedded in paraffin and cut at a thickness of 5 μm.

### 2.14 Histologic observation and analysis

Sections were stained for and observed under a light microscope (Eclipse E400; Nikon, Tokyo, Japan). Histological characteristics, such as pulp tissue disorganization (PTD), inflammatory cell infiltration (ICI) and dentin bridge formation (DBF), were evaluated according to modified versions of the ISO 10993 and 7405 standards as previously described that allows detailed evaluation of the changes in pulp tissue after direct pulp capping.^21^, ^22^ The scores of each sample were determined in a blinded manner by 3 observers.

### 2.15 Immunohistochemistry

The immunohistochemistry procedures were performed following the manufacturer's instructions (ZSGB‐Bio, Beijing, China). Sections were routinely dewaxed to water. After antigen retrieval and serum blocking, primary antibodies against FSHR (Santa Cruz Biotechnology, Inc, USA, 1:200), DSP (Santa Cruz Biotechnology, Inc, 1:100), OCN (Santa Cruz Biotechnology, Inc, USA, 1:200) and RUNX2 (Santa Cruz Biotechnology, Inc, 1:200) were incubated separately. The sections were then incubated with each corresponding biotinylated secondary antibody and stained with fresh DAB. The nuclei were stained with haematoxylin. For each specimen, positive cells from five randomly selected areas in the pulp were counted under a light microscope a 400× magnification.

### 2.16 Statistical analysis

The results of all measurements are demonstrated as the mean value ± standard deviation. GraphPad Prism 5.0 for Windows (GraphPad Software, Inc) was used for statistical analysis by one‐way ANOVA and post‐Tukey or Bonferroni multiple comparison tests. *P* < 0.05 was considered significant.

## 3 RESULTS

### 3.1 Characterization of DPSCs

**FIGURE 1:**
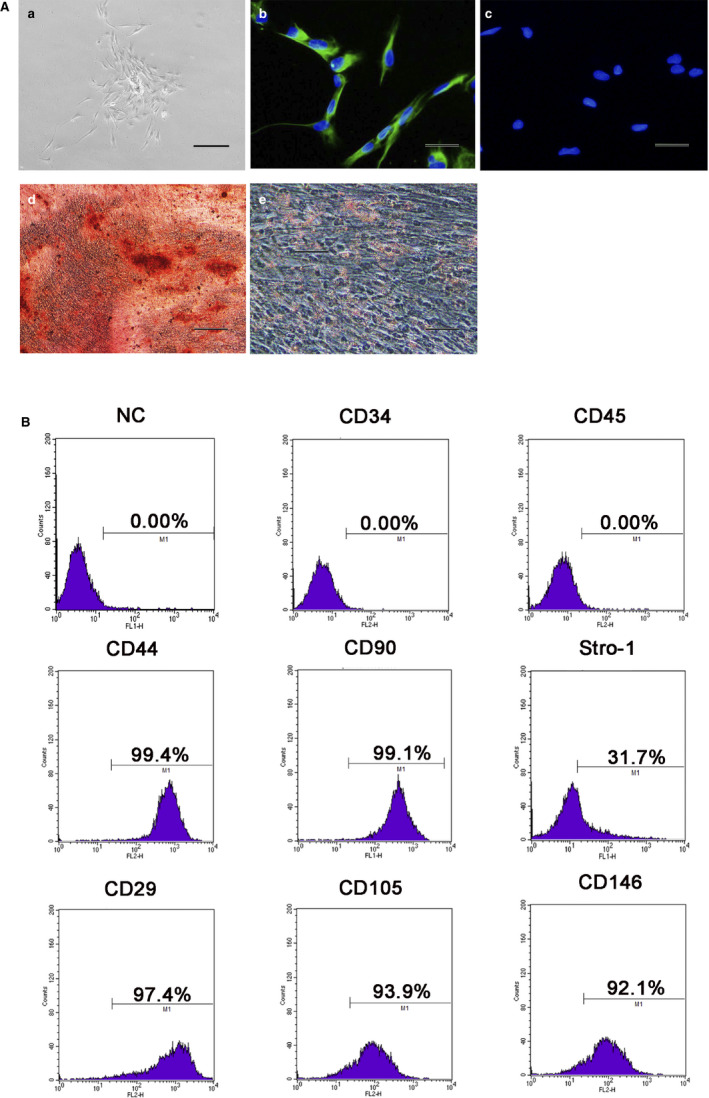
Characterization of human dental pulp stem cells (DPSCs). A, (a) Primary DPSCs showed fibroblast‐like morphology(Scale bar = 100 μm). Immunofluorescence showed positive staining of the DPSCs for vimentin (b) (Scale bar = 20 μm) and negative staining for keratin (c). (Scale bar = 20 μm) (d) Alizarin Red S positive staining of mineralized nodules in osteogenic induction of DPSCs. (Scale bar = 50 μm) (e) Positive Oil red O staining of the DPSCs after adipogenic induction. (Scale bar = 20 μm) B Flow cytometry detection of surface antigens of the DPSCs demonstrated negative expression of CD34 and CD45 and high expression of CD44, CD90, Stro‐1, CD29, CD105 and CD146

As previously described, DPSCs have a fibroblast‐like morphology. (Figure 1A(a)) Positive expression of vimentin (Figure 1A(b)) and negative expression of keratin (Figure 1A(c)) were detected in the DPSCs, indicating their mesenchymal origin.

Induction with different conditioned media, Alizarin Red S staining (Figure 1A(d)) and Oil Red O staining (Figure 1A(2)) demonstrated that the DPSCs possessed multiple differentiation capacities.

Surface antigens of the DPSCs were detected by flow cytometry. (Figure 1B) Hematopoietic markers (CD34 and CD45) were not expressed, while there was high expression of MSC markers (CD44, CD90, Stro‐1, CD29, CD105 and CD146).

### 3.2 Influence of FSH on the proliferation and apoptosis of the DPSCs

**FIGURE 2:**
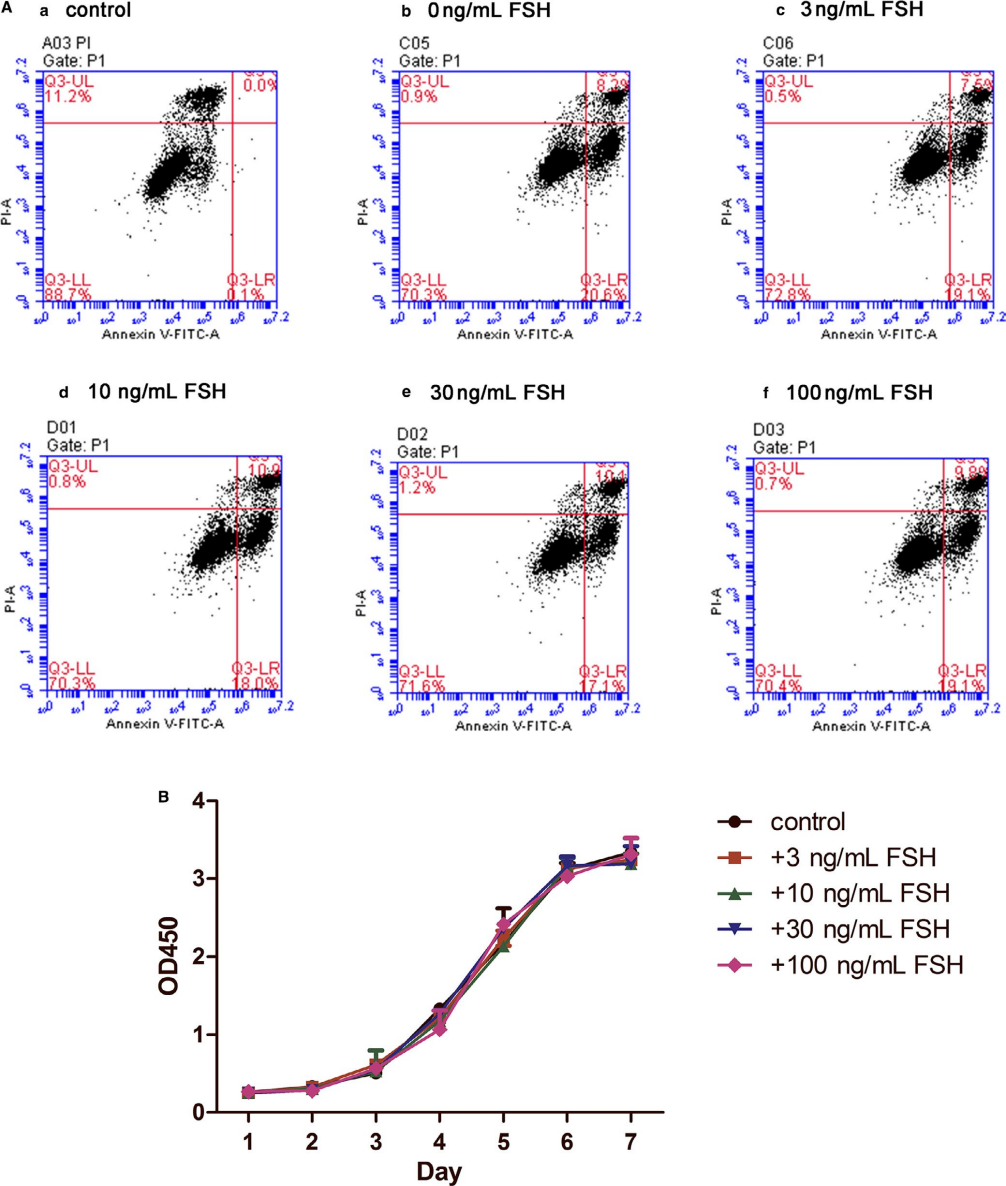
Effects of FSH on the apoptosis and proliferation of the DPSCs. A(a)‐(f), Cell apoptosis in the control and the 0‐100 ng/mL FSH groups by flow cytometry analysis. B, No significant proliferative differences by CCK8 were found in the 3‐100 ng/mL FSH groups compared with control group at 0‐7 d. (*P* > 0.05)

The proliferative capacity in the control group and the groups treated with different concentrations of FSH (3, 10, 30 ng/mL) were detected by CCK‐8 assays and were not significantly different at 0‐7 days (*P *> 0.05; Figure 2A). The effects of FSH on the apoptosis of the DPSCs were analysed using flow cytometry assays with an Annexin V‐FITC/PI Apoptosis Detection Kit. The cell apoptotic rates induced by different FSH concentrations showed no significant difference compared with that of the negative control. (*P *> 0.05; Figure 2B(a)‐(f)).

### 3.3 FSH decreased the odontogenic differentiation of the DPSCs

**FIGURE 3:**
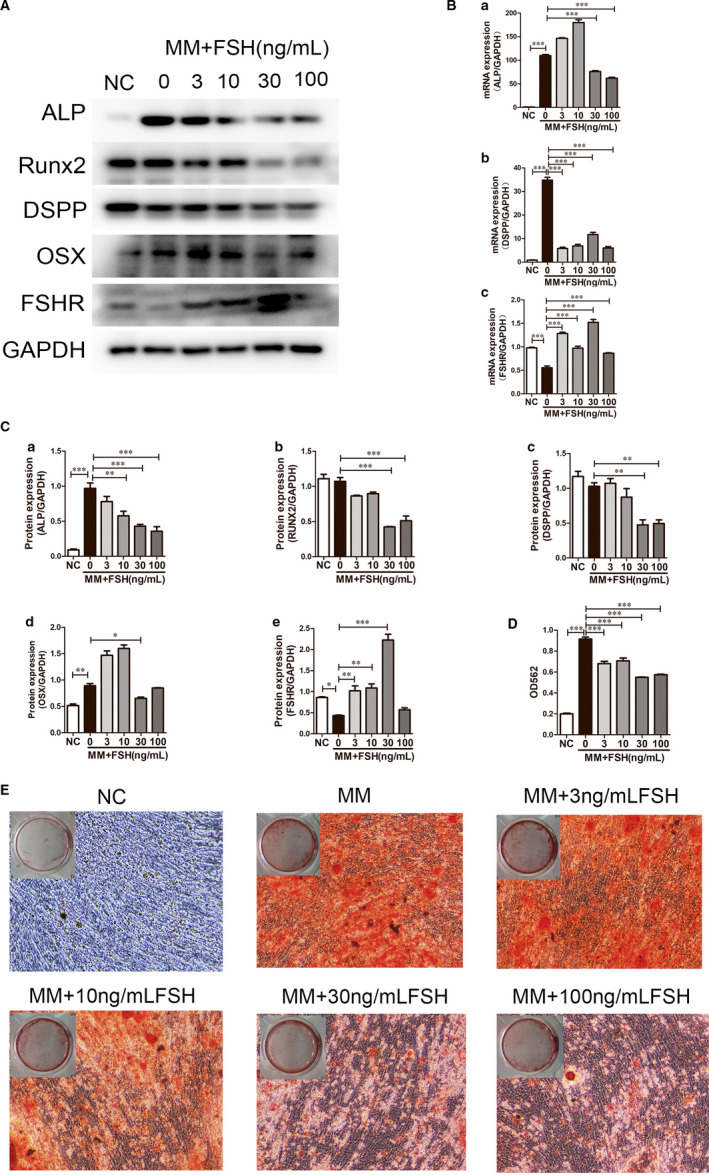
FSH decreased odonto/osteogenic differentiation of the DPSCs. A, Western blotting showed lower expression levels of ALP, Runx2, DSPP and OSX in the 30 ng/mL and 100 ng/mL FSH groups than the control group in odonto/osteogenic medium for 7 d. FSH significantly up‐regulated the FSHR protein expression, especially in the 30 ng/mL group. GAPDH was the internal control. B, mRNA expression levels of ALP, DSPP and FSHR levels of the DPSCs in the negative control and FSH‐treated groups (3‐100 ng/mL) in odonto/osteogenic medium for 3 d. Data are presented as the mean ± standard deviation, ***P < 0.0001. C, Grey scale analysis of the protein levels in the Western blotting results in A by ImageJ. Data are presented as the mean ± standard deviation, *P < 0.05, **P < 0.01, ***P < 0.0001. D, After odonto/osteogenic induction for 14 d, calcium contents in the 30 ng/mL and 100 ng/mL group were lower than those in the group without FSH treatment. Data are presented as the mean ± standard deviation. ***P < 0.0001. E, After odonto/osteogenic induction for 14 d, Alizarin Red staining of the mineralization nodules showed fewer calcified nodules in the 10‐100 ng/mL FSH‐treated groups, especially the 30 and 100 ng/mL groups, than in the control group

To investigate whether FSH could directly act on the DPSCs, we performed real‐time PCR to assess the expression of FSHR mRNA in the DPSCs and to confirm the identity of the PCR product performed with DNA sequencing (data not shown). Real‐time PCR showed that 3, 10 and 30 ng/mL FSH significantly induced FSHR expression, especially 30 ng/mL, while 100 ng/mL showed no significant changes compared with the negative control (Figure 3B(c)). Western blotting showed that protein expression levels of FSHR were significantly up‐regulated by FSH application, especially 30 ng/mL (Figure 3A). The results demonstrated that the DPSCs could directly act as targets of FSH via the FSH receptor.

The influence of FSH on odontogenic differentiation was examined. Western blot analysis revealed that the protein levels of ALP, RUNX2, DSPP and OSX were the lowest in the 30 ng/mL FSH group (Figure 3A). Quantitative analysis of the protein bands normalized to GAPDH is depicted in Figure 3C. Quantitative real‐time PCR demonstrated that the expression levels of osteo/odontogenic‐related genes, such as ALP and DSPP, were significantly lower in the DPSCs cultured in mineralization‐inducing medium (MM) with FSH (30 ng/mL) for 3 days than in those cultured in control medium (Figure 3B(a,b)). In addition, fewer calcified nodules were found in the 30 ng/mL FSH + MM (mineralization‐inducing medium) group than in the MM group (Figure 3E). CPC assay further presented that the calcium concentration in the 30 ng/mL FSH + MM group was lower than that in the MM group (*P* < 0.05; Figure 3D).

### 3.4 FSH activated the JNK‐MAPK pathway in DPSCs

**FIGURE 4:**
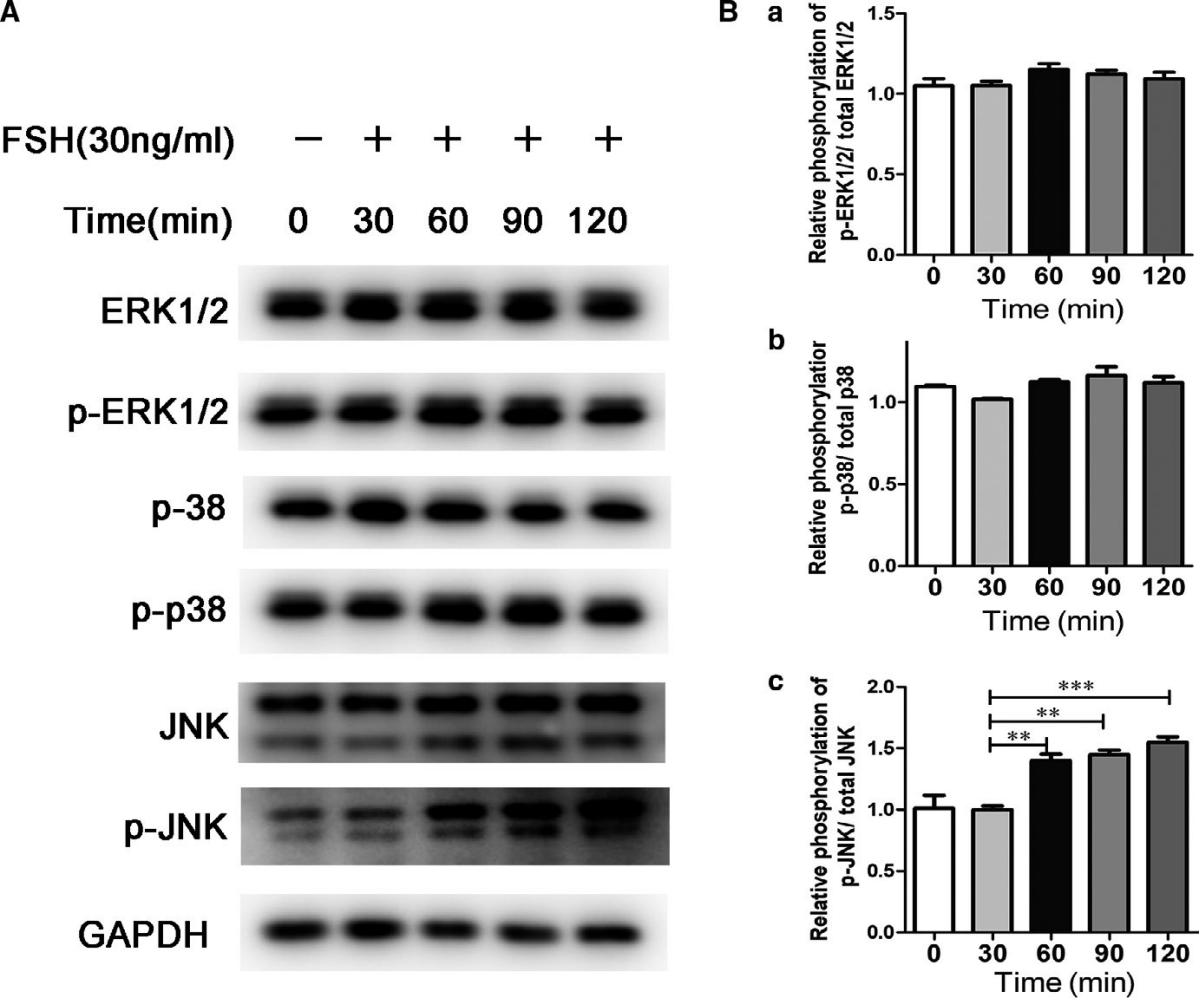
A concentration of 30 ng/mL FSH activated the JNK pathway in the DPSCs. A, Protein expressions of ERK1/2, p‐ERK1/2, p38, p‐p38, JNK and p‐JNK by Western blotting analysis. GAPDH was the internal control. B, Quantification analysis for the ratios of p‐ERK1/2/ ERK1/2 (a), p‐p38/pp8 (b) and p‐JNK/JNK (c) in A, Data are presented as the mean ± standard deviation,**P < 0.01, ***P < 0.0001

To explore the role of the MAPK pathways in the FSH‐mediated reduction of odontogenic differentiation of DPSCs, total proteins were extracted in the FSH‐treated (30 ng/mL) cells at 0, 30, 60, 90 and 120 minutes. Western blotting were performed to detect the protein levels of ERK, p‐ERK, P38, p‐P38, JNK and p‐JNK. In this study, FSH treatment did not affect the total ERK, P38 or JNK expression levels. ERK and p38 Phosphorylation did not change with time, and during FSH treatment, p‐JNK was gradually increased at 60 minutes and reached the highest level at 90 minutes (Figure 4A). The p‐JNK/JNK ratio indicated the activation of the JNK pathway after FSH treatment (Figure 4B).

### 3.5 Inhibition of the JNK‐MAPK pathways up‐regulated odonto/osteogenic differentiation of the FSH‐treated DPSCs

**FIGURE 5:**
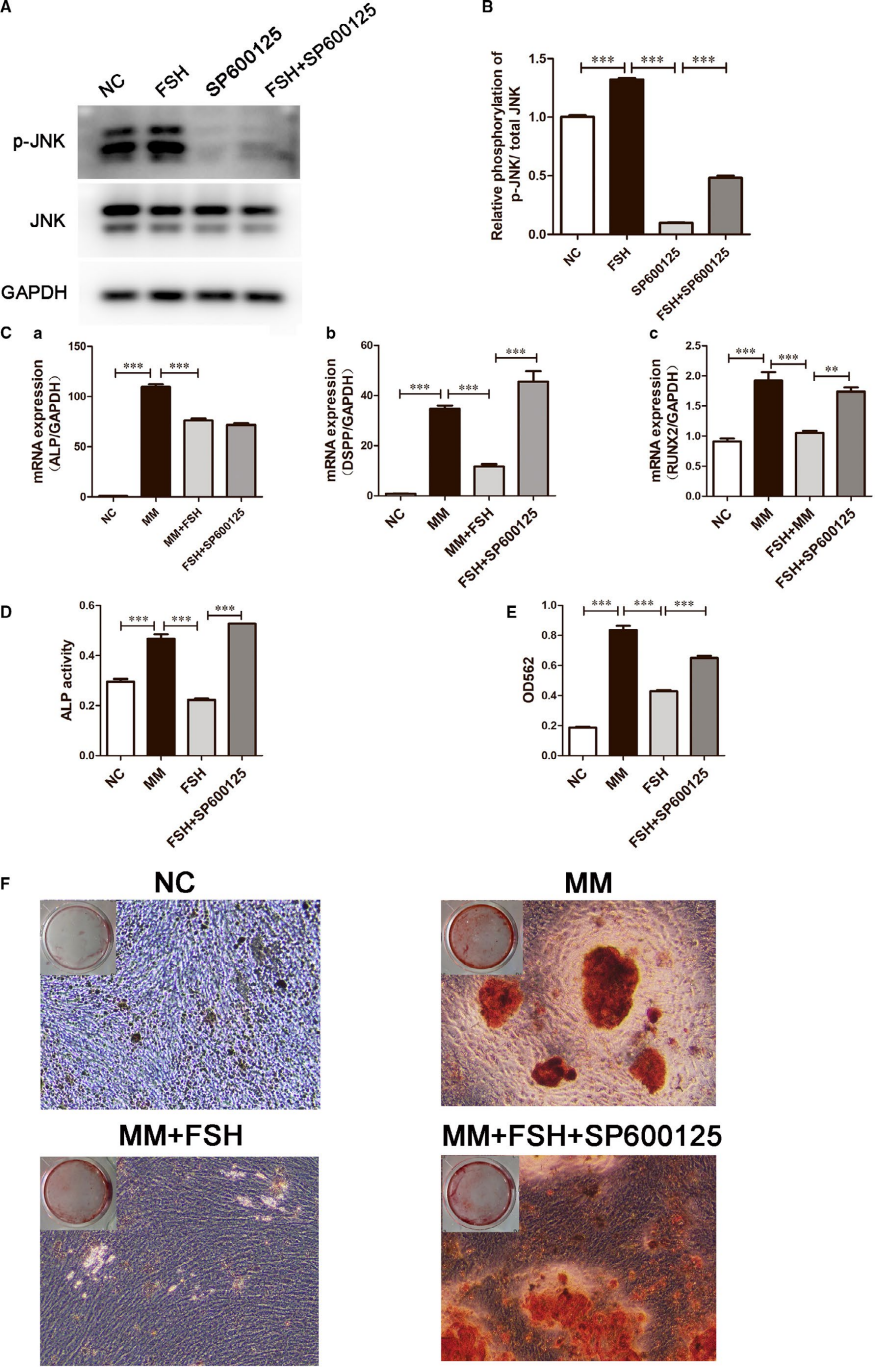
Effects of the JNK inhibitor on odonto/osteogenic differentiation of DPSCs treated with the FSH (30 ng/mL). A, Protein levels of p‐JNK and JNK at 60 min in the NC group, FSH group, SP600125 group and FSH + SP600125 group. GAPDH was the internal control. B, Grey scale analysis of the protein levels in the western blotting results in A by ImageJ. Data are presented as the mean ± standard deviation, ***P < 0.0001. C, The real‐time PCR expression levels of ALP (a), DSPP (b) and RUNX2 (c) in the FSH + SP600125 group were up‐regulated in comparison with the FSH group at day 3. Data are presented as the mean ± standard deviation, **P < 0.01, ***P < 0.0001. D, The ALP activities in the NC group, mineralization medium (MM) group, FSH group and FSH + SP600125 group were detected at day 7. Data are presented as the mean ± standard deviation, ***P < 0.0001. E, Calcium contents in the FSH + SP600125 group were higher than that in the FSH group at day 7. Data are presented as the mean ± standard deviation, ***P < 0.0001. F, Alizarin Red staining of the mineralized nodules in the NC group, mineralization medium (MM) group, MM + FSH group and MM + FSH + SP600125 group for 14 d. FSH, follicle‐stimulating hormone; DPSCs, dental pulp stem cells; ALP, alkaline phosphatase; JNK, c‐Jun N‐terminal kinases

To further clarify the role of the JNK‐MAPK signalling pathway in the odontogenic differentiation of the FSH‐treated DPSCs, 10 μmol/L SP600125 (a specific inhibitor of JNK) was added to suppress the activity of JNK. Prior to FSH (30 ng/mL) treatment, the DPSCs were incubated with SP600125 for 90 minutes. Western blotting assay was performed to detect the efficiency of SP600125. After administration of SP600125, p‐JNK was down‐regulated compared to the 60 minutes group (Figure 5A,B). Real‐time PCR presented that ALP, DSPP and RUNX2 mRNA expression levels were higher in FSH + SP600125 group compared with FSH group (Figure 5C). The ALP activity of the DPSCs in the FSH + SP600125 treatment group at day 7 was higher than that in the FSH treatment group (Figure 5D). Alizarin Red S staining showed significantly increased mineralization after 2 weeks odontogenic differentiation induction in FSH + SP600125 group compared to the FSH group (Figure 5F). The calcium concentration by CPC assay further presented that in the FSH + SP600125 group was higher than that in the FSH group (*P* < 0.05; Figure 3D).

### 3.6 FSH attenuated reparative dentin formation in rat molars

**FIGURE 6:**
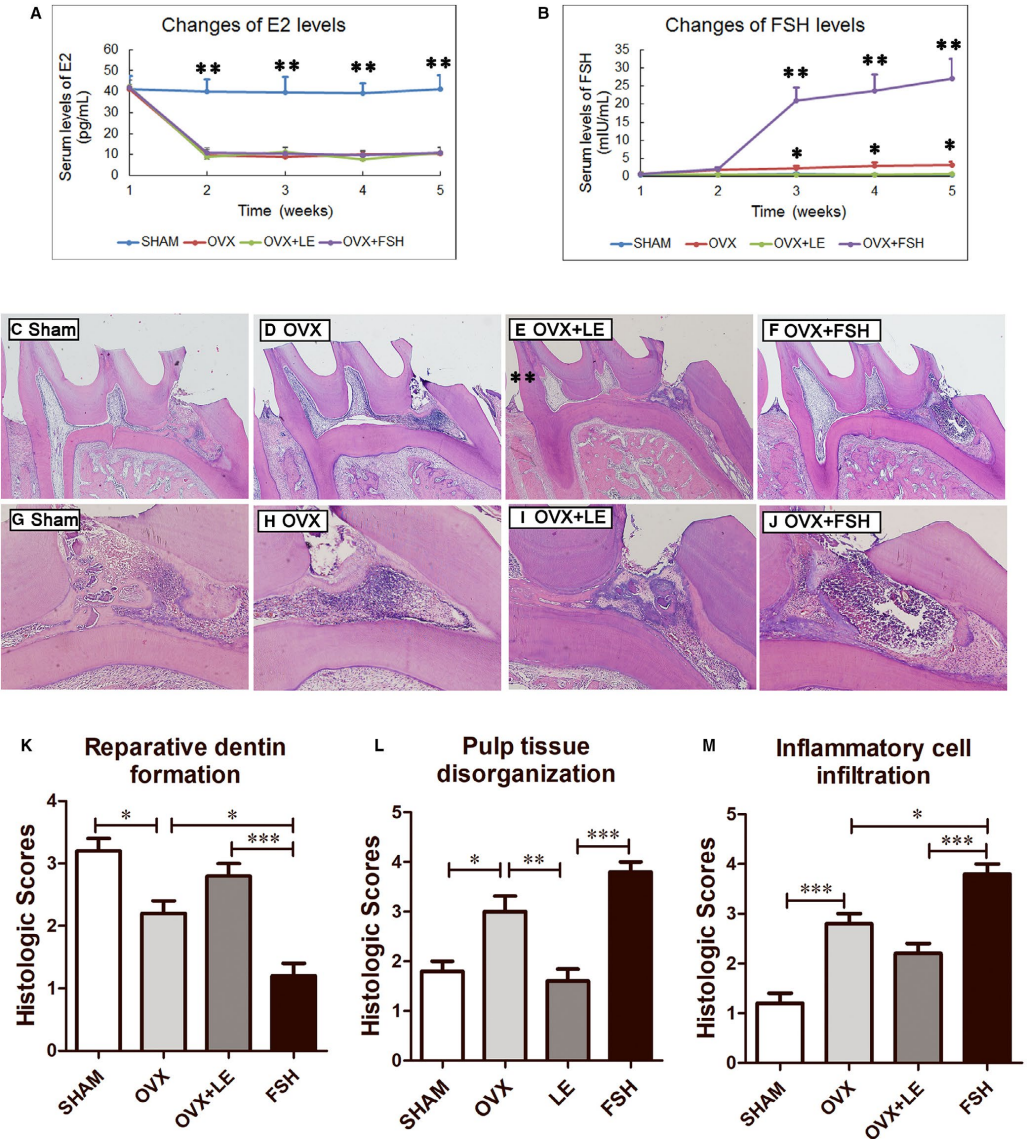
FSH impaired dental pulp healing after direct pulp capping in the OVX rats. A, The levels of E2 in the serum. Data are presented as the mean ± standard deviation. **P < 0.01 (Sham group vs OVX/OVX + LE/OVX + FSH group). B, Levels of FSH in the serum. Data are presented as the mean ± standard deviation. *P < 0.05 (Sham group vs OVX group; OVX group vs OVX + LE group), **P < 0.01 (OVX + FSH group vs Sham/ OVX/OVX + LE group). C‐J, Representative pictures of histologic features in each group at day 21. (C,G) Sham, (D,H) OVX, (E,I) OVX + LE, (F,J) OVX + FSH. (Haematoxylin‐eosin staining; original magnification, ×40 for C‐F, ×200 for G‐J). K‐M Summary of different categories of histologic features according to the scores. Data are presented as the mean ± standard deviation, *P < 0.05, **P < 0.01, ***P < 0.0001

The E2 and FSH levels in the rat serum were recorded every week. From 7 days after OVX operation, E2 levels in the serum decreased significantly in comparison with SHAM group(*P* < 0.01), with no significant changes found by use of LE and FSH(*P* > 0.05) (Figure 6A). FSH levels were significantly increased in OVX group. In FSH + LE group, FSH levels significantly were significantly lower than that in OVX group (*P* < 0.05). After injection of FSH, its serum levels ascended significant than those without FSH injection (*P* < 0.05) (Figure 6B).

The molars with filling loss and periapical lesions by X‐ray were excluded from the analysis of pulpal response. The histologic analysis is presented in Figure 6C‐J. On day 21, in the sham group, reparative dentin was directly observed at the injured region in the pulps capped with MTA. The adjacent pulp tissue appeared normal and free of inflammatory cells. (Figure 6C,G) Compared with those in the sham groups, the specimens in the OVX group exhibited less and poorly organized reparative dentin formation as well as stronger dental pulp inflammation (Figure 6D,H). LE application rescued the reparative dentin formation and weakened the dental pulp inflammation in the OVX rats (Figure 6 E,I). Reparative dentin formation was the lowest in the FSH treatment group, and inflammation below the dentinal bridge was the most severe in the FSH group compared with all other groups (Figure 6F,J). A summary of histopathological evaluation results is presented in Figure 6K‐M.

### 3.7 FSH weakened the expression of the mineralization‐related markers

**FIGURE 7:**
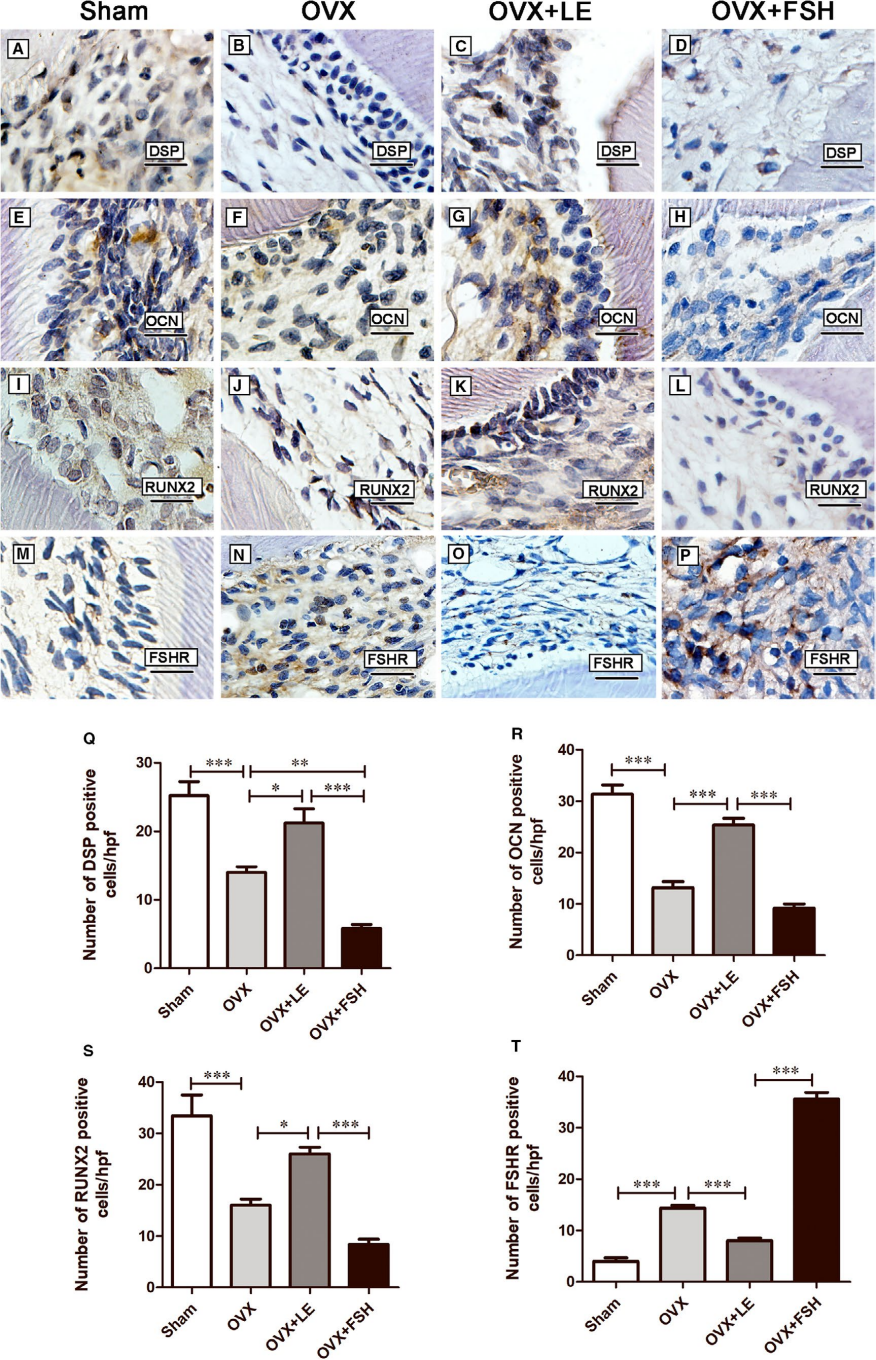
Immunohistochemical observation of mineralization‐related proteins and FSHR. A‐P, present the immunostaining for DSP (A‐D), OCN (E‐H), RUNX2 (I‐L) and FSHR (M‐P) in the dentin‐pulp complex. Q‐T shows the quantitative analysis of their histologic expression levels in each group. Scale bars = 50 μm. Data are presented as the mean ± standard deviation, *P < 0.05, **P < 0.01, ***P < 0.0001

The IHC results showed that expression DSP, OCN and RUNX2 was stronger in the cell‐rich zone of sham molars (Figure 7A,E,I,Q‐S) than in the cell‐rich zone of the OVX molars (Figure 7B,F,J,Q‐S) (*P* < 0.05). The number of DSP‐, OCN‐ and RUNX2‐positive cells was significantly reduced in the FSH treatment group (Figure 7D,H,L,Q‐S) compared with the non‐FSH treatment groups; the number of DSP‐, OCN‐ and RUNX2‐positive cells was significantly increased in the OVX + LE groups compared with the OVX group (Figure 7C,G,K,Q‐S) (*P* < 0.05).

The number of FSHR‐positive cells in the OVX group (Figure 7N,T) was larger than that in the sham group (Figure 7M,T). FSH treatment significantly increased the number of FSHR‐positive cells compared with those in the non‐FSH treatment groups (Figure 7P,T), which were significantly lower in the LE group than in the OVX group (Figure 7O,T). The majority of these FSHR‐positive cells were likely dental pulp fibroblasts, predominantly in the cell‐rich zone localized underneath the odontoblast layer.

## 4 DISCUSSION

Previous studies have mainly attributed the aetiology of diseases accompanying menopause solely to oestrogen deficiency. FSH is controlled by oestrogen levels through negative feedback. Traditionally, FSH was thought to be mainly targeting gonadal tissues, such as granulosa cells in the ovaries and Sertoli cells in the testis. Recently, these ideas are changing.^23^ In recent years, studies have shown that FSH directly affects skeletal remodelling during menopause ^16^ and inhibition of FSHβ rescues bone loss post‐ovariectomy.^17^, ^24^ FSH can increase the secretion of TNF‐α by granulocytes and macrophages in bone marrow, while FSH can promote the production of IL‐1, TNF‐α and IL‐6 by osteoclast precursor cells.^25^, ^26^ Blocking FSHβ can not only inhibit osteoclast activity and reduce the inflammatory response but also increase bone formation and osteoblast number.^17^ The influence of oestrogen deficiency on DPSCs has been elucidated. Oestrogen deficiency can down‐regulate the odonto/osteogenic capability of DPSCs both in vitro and in vivo, with no effect on proliferation.^10^ However, the effects of FSH on dentin and DPSCs have rarely been reported. Whether FSH could act on DPSCs remains unclear at present.

FSH exerts its biological action through its specific receptor, FSH receptor (FSHR), a type of G protein‐coupled receptors. FSHR was traditionally considered to be specifically expressed in gonadal tissues. In recent decades, tissues in addition to gonads, including bone, have been found to express FSHRs.^27^ In bone tissues, the activation of FSHR in osteoclasts can promote osteoclast formation and enhance osteoclast function. FSHRs were expressed on bone marrow mesenchymal stem cells (BMSCs), while their expression was down‐regulated in mature osteoblasts, indicating that FSHRs may negatively drive MSC differentiated into osteoblasts.^16^ Application of an FSH antibody could induce osteoblast colonies formation. Similarly, osteoblast colonies cultured from FSHR^‐/‐^ mice were more than those in wild type.^24^ DPSCs are important mesenchymal stem cells in dentin–pulp complex, possessing many similarities to BMSCs. In our study, we found that FSHR was expressed in the DPSCs. Application of FSH, especially at a concentration of 30 ng/mL, significantly up‐regulated the expression levels of FSHR in the DPSCs. We also found that the FSHR expression levels declined during odonto‐osteogenic differentiation of the DPSCs.

The proliferative and differentiated abilities of DPSCs are very important features in regeneration of dentin–pulp complex. In our study, a potential impact of FSH on the proliferation of DPSCs was investigated. We found that 3, 10, 30 and 100 ng/mL FSH did not affect the proliferation of DPSCs. No obvious changes in the effects of FSH on the apoptotic rate of the DPSCs were found. FSH (30 ng/mL) significantly decreased the ALP activity, mineralization capacity and odonto/osteogenic potential of the cells. FSH could down‐regulate the protein levels of osteo/odontogenic‐related genes, including DSPP, ALP, RUNX2 and OSX. Runx2 belongs to the Runx family of transcription factors, which is a key regulator of osteoblast differentiation.^28^ Runx2 is expressed in osteoblast lineage cells and chondrocytes.^29^ In Runx2^−/−^ mice, intramembranous and endochondral bone formation are both completely interrupted.^30^ Runx2 significantly stimulated calcium accumulation and alkaline phosphatase activity in dental pulp stem cells (DPSCs).^31^ Dentin sialophosphoprotein (DSPP), a chimeric extracellular matrix protein, is expressed in teeth, predominantly by odontoblasts.^32^ This protein is processed by proteases into three protein products: dentin sialoprotein (DSP), dentin glycoprotein (DGP) and dentin phosphoprotein (DPP). DSPP is important in dentin biomineralization, the mutations of which interrupt mineralization homeostasis during odontoblast differentiation.^33^ Osx is an osteoblast‐specific transcription factor required for bone formation and tooth development.^34^ Osx has been identified to be associated with odontoblast differentiation.^35^

MAPK is a broad‐based serine/threonine protein kinase and plays an important role in signal transduction of cell proliferation and differentiation in DPSCs.^36^, ^37^ MAPK was mainly activated by two different types of receptors on cell membrane, G protein‐coupled receptors and receptor tyrosine kinases.^38^ MAPKs are divided into three subtypes: extracellular signal‐regulated kinase (ERK), p38 and Jun N‐terminal kinases/stress‐activated protein kinases (JNK). FSH binds to FSHR, a kind of G protein‐coupled receptor. FSH has been reported to activate MAPK pathways in ovarian cells. In granulosa cells, FSH could activate p38, ERK1/2 and JNK via FSHR, then regulating cell proliferation, survival and steroidogenesis.^39^, ^40^, ^41^ To clarify the mechanism of FSH regulating odonto/osteogenic differentiation of DPSCs, the MAPK pathway was chosen and investigated. In this study, FSH promoted the phosphorylation of JNK, indicating the activation of the JNK pathway in the FSH‐treated DPSCs during osteo/odontogenic differentiation. To further elucidate the functions of JNK cascade in the osteo/odontogenic differentiation of FSH‐treated DPSCs, we applied a JNK inhibitor. Blocking JNK rescued the inhibitory effects of FSH on the osteo/odontogenic differentiation of the DPSCs. Our results suggested that FSH induced the phosphorylation of the JNK‐MAPK pathway during suppression of the osteo/odontogenic differentiation of the DPSCs.

The ovariectomized (OVX) rat has been applied widely as menopausal model, as it has been validated to show oestrogen deficiency.^42^ However, only ovariectomy model was unable to highlight the roles of FSH. Our group administered FSH and its inhibitor based on OVX model to study the independent role of FSH.^18^, ^20^ No obvious changes were found in E2 levels of OVX groups after FSH and LE application. Furthermore, significant changes were found in FSH levels in OVX + FSH and OVX + LE groups compared with OVX group. Based on this rat model, we combined a direct pulp‐capping model to test the role of FSH in dental pulp healing. Herein, as previously observed, the formation of dentin bridges was observed beneath the perforation of dental pulps in the Sham group.^19^ Histologically, higher rates of inflammation and less calcified bridge formation in the OVX groups. This condition was more severe in the FSH groups and alleviated in the LE groups. We also gained insights into the role of some mineralization‐related proteins, such as DSP, OCN and RUNX2. In this study, the OVX group presented weaker expression of several odonto/osteogenic proteins in the dentin‐pulp complex than the sham group, which was in accordance with a previous study.^11^ Administration of FSH further weakened the expression of odonto/osteogenic proteins, and in the LE groups, their expression levels were stronger than those in the OVX groups. Since the cell‐rich zone in the dentin‐pulp complex mainly contains pulp fibroblasts and DPSCs, the down‐regulation of these odonto/osteogenic proteins may indicate that the differentiation rate of the DPSCs in the OVX groups was decreased compared with that in the sham groups. The FSH‐treated groups and the LE groups presented the opposite results, indicating that FSH may inhibit the differentiation of the DPSCs. Therefore, these results suggested that FSH, independent of oestrogen, can impair the formation of reparative dentin at the injury site of direct pulp capping.

In summary, this study showed that FSH did not influence the proliferation of human DPSCs. FSH, especially at a concentration of 30 ng/mL, inhibited osteo/odontogenic differentiation of the DPSCs via modulating the JNK‐MAPK pathway. Moreover, an in vivo study further confirmed that FSH could block the reparative dentine formation in the OVX rats. Although further studies are required, our findings suggest that FSH may play a role in the DPSCs as a negative regulator of mineralization, which may be a crucial factor for clinical pulp healing and regeneration during menopause.

## CONFLICT OF INTEREST

The authors deny any conflicts of interest.

## AUTHOR CONTRIBUTION

**Hua Qian:** Data curation (lead); Formal analysis (lead); Funding acquisition (lead); Investigation (lead); Methodology (lead); Project administration (lead); Validation (lead); Visualization (lead); Writing‐original draft (lead); Writing‐review & editing (lead). **Xiaoyue Guan:** Methodology (supporting).

## Supporting information

Table S1

## Data Availability

The data that support the findings of this study are openly available in [repository name, eg “figshare”] at http://doi.org/[doi], reference number [reference number].
