# Supplementary material for: Follicle‐stimulating hormone impairs dental pulp stem cells odontogenic differentiation
Source: J Cell Mol Med. 2020 Jul 28;24(18):10621–35. doi: 10.1111/jcmm.15681 (PMC7521281; doi:10.1111/jcmm.15681)
Supplement: Supplementary file 1 — Table S1 [file JCMM-24-10621-s001.docx]

Supplementary Table

Primer sequences for real time PCR

| Gene | Forward primer (5’-3’) | Reverse primer(5’-3’) |
| --- | --- | --- |
| ALP | ACCACCACGAGAGTGAACCA | CGTTGTCTGAGTACCAGTCCC |
| DSPP | TTTGGGCAGTAGCATGGGC | CCATCTTGGGTATTCTCTTGCCT |
| FSHR | ATCTTAAGAAGCTGAGGGCCAGGT | CAGTTTGCAAAGGCACAGCAATGG |
| RUNX2 | TGGTTACTGTCATGGCGGGTA | TCTCAGATCGTTGAACCTTGCTA |
| GAPDH | CAAGAAGGTGGTGAAGCAGG | AAAGTGGTCGTTGAGGGCA |
